# Dataset describing the amino acid catabolism of *Thermoanaerobacter* strain AK85: The influence of culture conditions on end product formation

**DOI:** 10.1016/j.dib.2019.103938

**Published:** 2019-04-23

**Authors:** Sean Michael Scully, Johann Orlygsson

**Affiliations:** University of Akureyri, Faculty of Natural Resource Sciences, Borgir 2 v/ Norðurslóð, 600 Akureyri, Iceland

**Keywords:** Thermophiles, Branched-chain amino acids and alcohols, Thiosulfate

## Abstract

The dataset describes the catabolism of the 20 proteogenics amino acids and their end products by *Thermoanaerobacter* strain AK85 under different electron scavenging conditions with an emphasis on the branched-chain amino acids as reported in Scully and Orlygsson, 2019.

**Table:** Specifications table

Subject area	Biology
More specific subject area	Microbiology
Type of data	Raw data
How data was acquired	GC-FID, Perkin Elmer Clarus 580 GC-TCD, Perkin Elmer Autosystem XL UV–Visible Spectroscopy, Bioscreen C (GrowthCurves Ltd, Finland) and Shimadzu UV-1800 UV–Visible Spectrometer
Data format	Table
Experimental factors	Substrate, growth temperature, initial pH, different liquid-gas phase ratio, different initial thiosulfate concentration.
Experimental features	Data shows fermentation products from amino acids as single substrates as well as with addition of thiosulfate to the culture. Effect of various environmental factors were investigated for the formation of a mixture of branched-chain fatty acids and branched-chain alcohols from branched-chain amino acids; pH, temperature, initial thiosulfate concentrations, different liquid-gas phase ratios. A kinetic experiment of all three branched-chain amino acids are also presented.
Data source location	University of Akureyri, Faculty of Natural Research Science, Borgir, Nordurslod 2, 600 Akureyri, Iceland.
Data accessibility	*The data is with this article*
Related research article	Scully and Orlygsson, 2019 [1]

**Table:** 

**Value of the data** • The data presents end products from the fermentation of 20 proteogenic amino acids with and without the use of a thiosulfate as an electron scavenging system by Thermoanaerobacter strain AK85, a thermophilic anaerobe isolated a hot spring in Iceland • The data set shows the influence of culture parameters on the fermentation of a branched-chain amino acid, L-isoleucine, in batch culture, as well as a kinetic experiment showing the formation of branched-chain fatty acids and alcohols from branched-chain amino acids • Could be a useful for comparing the amino acid catabolism of other mesophilic and thermophilic anaerobes producing higher-value C4 and C5 alcohols.

## 1 Data

*Thermoanaerobacter* strain AK85 ferments serine to a predominantly to acetate (but also minor amounts of ethanol) with and without an added electron scavenging system [1]. The branched-chain amino acids (BCAAs) valine, leucine, and isoleucine, are catabolized to their corresponding branched-chain fatty acids (BCFAs) when co-cultured with a hydrogenotrophic methanogen and to a mixture of their BCFA and branched-chain alcohols (BCOH) in the presence of 40 mM of thiosulfate [1]. Additionally, threonine is degraded mainly to acetate but also some ethanol was produced under thiosulfate and methanogenic conditions. Other amino acid were not degraded.

The influence of various culture parameters were investigated in batch culture using isoleucine as a model compound for BCAA catabolism. Table 1 details the influence of initial pH on end product formation for isoleucine while Table 2 displays end product formation as a function of cultivation temperature between 50 and 85 °C. The influence of liquid-gas (L-G) phase ratio on end product formation is presented in Table 3 and the effect of initial thiosulfate concentrations between 0 and 60 mM are shown in Table 4. To investigate the effect of both L-G and initial thiosulfate concentration, a two variable experiment was conducted using isoleucine as a substrate (Table 5). Detailed kinetic experiments were performed over a period of 7 days using the valine, isoleucine, and leucine (individually) as shown in Table 6, Table 7, Table 8, respectively.

**Table 1:** Influence of pH on the fermentation of isoleucine (20 mM) and thiosulfate (20 mM) by *Thermoanaerobacter* strain AK85 after 14 days of cultivation. Values represent the average of triplicate measures ± standard deviation.

Initial pH	Analyte (mmol/L)								Ile degraded (%)	Carbon balance (%)	OD	pH
	Ethanol	2-methyl-1-butanol	Acetate	2-methyl-1-butyrate	H_2_	H_2_S	S_2_O_3_	Ile				
4.0	0.51 ± 0.10	2.34 ± 0.90	3.34 ± 0.39	4.34 ± 0.68	0.82 ± 0.27	3.04 ± 0.21	5.34 ± 0.37	11.85 ± 0.83	40.8	92.7	0.05 ± 0.00	4.3 ± 0.2
4.5	2.17 ± 0.24	8.40 ± 1.57	2.57 ± 0.24	6.64 ± 1.31	1.16 ± 0.34	2.68 ± 0.33	<0.50	5.34 ± 1.01	73.3	101.9	0.13 ± 0.01	4.9 ± 0.1
5.0	3.78 ± 0.13	10.44 ± 1.04	2.66 ± 0.34	7.01 ± 1.47	1.70 ± 0.45	2.48 ± 0.39	<0.50	1.83 ± 0.43	90.9	96.4	0.17 ± 0.03	5.2 ± 0.0
5.5	3.89 ± 0.32	12.19 ± 0.47	2.41 ± 0.12	7.46 ± 1.24	1.27 ± 0.48	2.02 ± 0.45	<0.50	0.57 ± 0.32	97.2	101.1	0.17 ± 0.02	5.8 ± 0.1
6.0	4.41 ± 0.34	11.34 ± 1.78	2.67 ± 0.27	7.91 ± 1.38	1.43 ± 0.27	2.37 ± 0.41	<0.50	1.68 ± 0.71	91.6	104.7	0.18 ± 0.01	6.6 ± 0.2
6.5	4.36 ± 0.27	11.24 ± 0.84	2.57 ± 0.21	7.68 ± 0.98	1.07 ± 0.17	2.47 ± 0.20	<0.50	1.24 ± 0.50	93.8	100.8	0.19 ± 0.02	6.8 ± 0.0
7.0	4.62 ± 0.17	13.47 ± 1.23	2.47 ± 0.16	7.81 ± 1.77	1.24 ± 0.37	2.33 ± 0.31	<0.50	0.89 ± 0.36	95.6	110.9	0.19 ± 0.01	7.3 ± 0.1
7.5	4.67 ± 0.16	12.47 ± 0.44	2.85 ± 0.07	7.61 ± 0.64	0.78 ± 0.31	2.46 ± 0.13	<0.50	1.51 ± 0.33	92.5	108.0	0.21 ± 0.02	7.6 ± 0.1
8.0	4.40 ± 0.24	11.78 ± 1.32	2.29 ± 0.18	6.67 ± 1.34	1.01 ± 0.37	2.64 ± 0.37	<0.50	3.53 ± 0.85	82.4	109.9	0.13 ± 0.05	8.2 ± 0.1
8.5	3.87 ± 0.20	12.10 ± 0.89	2.07 ± 0.42	6.70 ± 1.37	1.23 ± 0.47	3.71 ± 0.25	<0.50	3.34 ± 0.91	83.3	110.7	0.12 ± 0.03	8.8 ± 0.0
9.0	3.01 ± 0.13	6.01 ± 1.67	1.37 ± 0.24	4.78 ± 1.59	0.72 ± 0.22	4.21 ± 0.36	<0.50	12.21 ± 2.87	39.0	115.0	0.09 ± 0.01	9.3 ± 0.1

**Table 2:** Influence of temperature on the fermentation of isoleucine (20 mM) and thiosulfate (20 mM) by *Thermoanaerobacter* strain AK85 after 14 days of cultivation. Values represent the average of triplicate measures ± standard deviation.

Temp (°C)	Analyte (mmol/L)								Ile degraded (%)	Carbon balance (%)	OD	pH
	Ethanol	2-methyl-1-butanol	Acetate	2-methyl-1-butyrate	H_2_	H_2_S	S_2_O_3_	Ile				
50	3.07 ± 0.10	6.37 ± 0.17	1.87 ± 0.43	5.02 ± 1.76	0.27 ± 0.17	0.87 ± 0.33	<0.50	14.27 ± 2.57	28.7	128.3	0.08 ± 0.01	7.2 ± 0.1
55	3.78 ± 0.13	10.44 ± 1.04	2.60 ± 0.35	6.99 ± 0.83	0.43 ± 0.21	1.78 ± 0.27	<0.50	5.55 ± 0.54	72.3	114.9	0.09 ± 0.02	7.3 ± 0.2
60	4.67 ± 0.16	12.21 ± 0.28	2.48 ± 0.17	7.63 ± 0.49	0.78 ± 0.31	2.57 ± 0.53	<0.50	1.30 ± 0.41	93.5	105.7	0.18 ± 0.03	7.4 ± 0.0
65	4.61 ± 0.26	12.47 ± 0.44	2.85 ± 0.12	7.67 ± 0.64	1.03 ± 0.28	2.46 ± 0.13	<0.50	1.84 ± 0.27	90.8	109.9	0.20 ± 0.02	7.3 ± 0.1
70	4.35 ± 0.37	12.34 ± 0.47	2.67 ± 0.21	7.91 ± 0.24	1.45 ± 0.39	3.71 ± 0.22	<0.50	2.51 ± 0.47	87.5	113.8	0.20 ± 0.02	7.4 ± 0.0
75	4.11 ± 0.24	11.87 ± 0.87	2.47 ± 0.34	8.07 ± 0.37	1.21 ± 0.21	1.56 ± 0.46	<0.50	3.29 ± 0.34	83.6	116.2	0.16 ± 0.01	7.3 ± 0.2
80	1.78 ± 0.13	5.41 ± 1.04	2.66 ± 0.30	3.17 ± 0.71	0.14 ± 0.07	1.86 ± 0.17	<0.50	15.28 ± 1.24	23.6	119.3	0.10 ± 0.02	7.4 ± 0.1
85	0.34 ± 0.10	1.11 ± 0.47	2.47 ± 0.22	1.13 ± 0.33	0.00 ± 0.00	0.17 ± 0.10	<0.50	19.55 ± 0.57	2.3	109.0	0.05 ± 0.01	7.2 ± 0.2

**Table 3:** Influence of liquid-gas phase ratio concentration on the fermentation of isoleucine (20 mM) and thiosulfate (20 mM|) by *Thermoanaerobacter* strain AK85. Values represent the average of triplicate measures ± standard deviation.

L-G	Analyte (mmol/L)								Ile degraded (%)	Carbon balance (%)	OD	pH
	Ethanol	2-methyl-1-butanol	Acetate	2-methyl-1-butyrate	H_2_	H_2_S	S_2_O_3_	Ile				
0.05	3.32 ± 0.15	8.41 ± 0.51	1.99 ± 0.04	12.25 ± 0.14	0.47 ± 0.11	1.20 ± 0.02	<0.50	0.56 ± 0.17	97.2	106.1	0.22 ± 0.02	7.3 ± 0.2
0.34	4.07 ± 0.40	10.87 ± 0.66	2.38 ± 0.21	7.31 ± 0.56	1.01 ± 0.35	1.88 ± 0.47	<0.50	0.64 ± 0.27	96.8	94.1	0.24 ± 0.03	7.1 ± 0.1
0.98	4.62 ± 0.17	13.47 ± 1.23	2.47 ± 0.16	7.81 ± 1.77	1.24 ± 0.37	2.33 ± 0.31	<0.50	0.79 ± 0.14	96.1	110.4	0.19 ± 0.01	7.4 ± 0.1
2.08	4.27 ± 0.44	12.17 ± 0.87	2.55 ± 0.27	8.24 ± 0.48	1.47 ± 0.28	2.78 ± 0.27	<0.50	0.23 ± 0.05	98.9	103.2	0.19 ± 0.01	7.2 ± 0.1
5.4	2.89 ± 0.09	6.17 ± 1.33	2.79 ± 0.20	8.62 ± 0.28	0.34 ± 0.02	3.32 ± 0.75	<0.50	2.15 ± 0.43	89.3	84.7	0.17 ± 0.01	7.3 ± 0.0

**Table 4:** Influence of thiosulfate concentration on the fermentation of isoleucine (20 mM) by *Thermoanaerobacter* strain AK85 after 14 days of cultivation. Values represent the average of triplicate measures ± standard deviation.

[S_2_O_3_] (mM)	Analyte (mmol/L)								Ile degraded (%)	Carbon balance (%)	OD	pH
	Ethanol	2-methyl-1-butanol	Acetate	2-methyl-1-butyrate	H_2_	H_2_S	S_2_O_3_	Ile				
0	2.39 ± 0.37	1.70 ± 0.56	1.82 ± 0.08	3.23 ± 0.01	2.40 ± 0.18	2.33 ± 0.31	<0.50	11.14 ± 2.74	44.3	80.3	0.19 ± 0.02	7.2 ± 0.1
10	3.96 ± 0.57	4.93 ± 1.05	1.93 ± 0.05	3.16 ± 0.26	1.52 ± 0.35	2.35 ± 2.16	<0.50	10.41 ± 0.68	48.0	92.5	0.20 ± 0.01	7.3 ± 0.1
20	4.53 ± 0.13	13.24 ± 1.14	2.34 ± 0.12	7.49 ± 0.22	1.14 ± 0.24	2.33 ± 0.31	<0.50	0.78 ± 0.24	96.1	107.5	0.20 ± 0.09	7.1 ± 0.0
30	4.01 ± 0.24	8.47 ± 0.74	2.81 ± 0.20	10.17 ± 0.34	0.67 ± 0.31	2.56 ± 0.47	<0.50	0.25 ± 0.07	98.8	94.5	0.22 ± 0.05	7.4 ± 0.2
40	2.75 ± 0.16	6.55 ± 0.70	3.07 ± 0.23	9.10 ± 1.11	0.86 ± 0.24	2.69 ± 0.87	<0.50	1.49 ± 0.18	92.6	85.7	0.21 ± 0.02	7.3 ± 0.1
50	2.46 ± 0.06	5.40 ± 0.51	3.40 ± 0.24	11.07 ± 1.40	0.21 ± 0.14	4.01 ± 0.44	<0.50	0.76 ± 0.05	96.2	86.2	0.25 ± 0.04	7.6 ± 0.3
60	2.20 ± 0.32	4.84 ± 0.72	3.70 ± 0.28	12.40 ± 0.77	0.19 ± 0.04	4.86 ± 0.49	<0.50	0.25 ± 0.11	98.7	87.5	0.26 ± 0.07	7.5 ± 0.2

**Table 5:** Influence of liquid-gas phase ratio and initial thiosulfate concentration on the fermentation of isoleucine (20 mM) by *Thermoanaerobacter* strain AK85. Values represent the average of triplicate measures ± standard deviation.

Substrate	Thiosulfate (mM)	L-G	Analyte (mmol/L)								% AA degraded	Carbon balance (%)	OD	pH
			Ethanol	2-methyl-1-butanol	Acetate	2-methyl-1-butyrate	H_2_	H_2_S	S_2_O_3_	Ile				
Control (YE)	0	0.05	2.71 ± 0.23	<0.50	2.13 ± 0.20	<0.50	0.54 ± 0.17	<0.50	ND	ND	ND	ND	0.17 ± 0.01	6.9 ± 0.1
	10	0.05	3.53 ± 0.34	<0.50	3.00 ± 0.17	<0.50	0.46 ± 0.08	<0.50	<0.50	ND	ND	ND	0.21 ± 0.01	7.1 ± 0.2
	20	0.05	3.66 ± 0.26	<0.50	2.18 ± 0.27	<0.50	0.47 ± 0.11	<0.50	<0.50	ND	ND	ND	0.22 ± 0.02	7.2 ± 0.1
	40	0.05	2.49 ± 0.10	<0.50	2.72 ± 0.14	<0.50	0.35 ± 0.17	<0.50	<0.50	ND	ND	ND	0.39 ± 0.04	6.8 ± 0.1
	0	0.98	2.13 ± 0.37	<0.50	1.63 ± 0.04	<0.50	2.40 ± 0.18	<0.50	ND	ND	ND	ND	0.19 ± 0.02	7.0 ± 0.1
	10	0.98	3.62 ± 0.12	<0.50	2.18 ± 0.23	<0.50	1.52 ± 0.35	<0.50	<0.50	ND	ND	ND	0.20 ± 0.01	6.8 ± 0.0
	20	0.98	3.76 ± 0.28	<0.50	3.26 ± 0.19	<0.50	1.14 ± 0.24	<0.50	<0.50	ND	ND	ND	0.19 ± 0.01	6.7 ± 0.1
	40	0.98	3.10 ± 0.34	<0.50	3.37 ± 0.27	<0.50	0.86 ± 0.24	<0.50	<0.50	ND	ND	ND	0.21 ± 0.04	6.7 ± 0.2
	0	5.4	3.97 ± 0.28	<0.50	1.93 ± 0.14	<0.50	2.27 ± 0.00	<0.50	ND	ND	ND	ND	0.16 ± 0.03	6.9 ± 0.1
	10	5.4	4.03 ± 0.24	<0.50	2.45 ± 0.26	<0.50	1.05 ± 0.20	<0.50	<0.50	ND	ND	ND	0.15 ± 0.02	6.8 ± 0.1
	20	5.4	3.15 ± 0.25	<0.50	2.61 ± 0.26	<0.50	0.34 ± 0.02	<0.50	<0.50	ND	ND	ND	0.17 ± 0.02	6.8 ± 0.3
	40	5.4	2.9 ± 0.16	<0.50	3.45 ± 0.17	<0.50	1.12 ± 0.20	<0.50	<0.50	ND	ND	ND	0.14 ± 0.0	6.6 ± 0.2
Isoleucine														
	0	0.05	2.93 ± 0.23	2.90 ± 0.06	1.84 ± 0.01	3.04 ± 1.01	0.54 ± 0.17	<0.50	<0.50	14.24 ± 1.32	26.4	103.3	0.17 ± 0.01	7.2 ± 0.1
	10	0.05	3.72 ± 0.08	11.56 ± 0.67	2.00 ± 0.05	5.44 ± 0.12	0.46 ± 0.08	0.61 ± 0.34	<0.50	2.85 ± 0.43	85.8	99.3	0.21 ± 0.01	7.6 ± 0.2
	20	0.05	3.32 ± 0.15	8.41 ± 0.51	1.99 ± 0.04	4.28 ± 0.14	0.47 ± 0.11	2.33 ± 0.31	<0.50	6.21 ± 0.63	69.00	94.5	0.22 ± 0.02	7.5 ± 0.2
	40	0.05	2.71 ± 0.07	6.93 ± 0.42	2.74 ± 0.31	10.29 ± 1.60	0.35 ± 0.17	2.69 ± 0.87	<0.50	1.85 ± 0.43	90.8	95.3	0.39 ± 0.04	7.6 ± 0.1
	0	0.98	2.39 ± 0.37	2.70 ± 1.11	1.82 ± 0.08	3.23 ± 0.01	2.40 ± 0.18	<0.50	<0.50	13.35 ± 2.17	24.4	105.3	0.19 ± 0.02	7.0 ± 0.2
	10	0.98	3.96 ± 0.57	4.93 ± 1.05	1.93 ± 0.05	3.16 ± 0.26	1.52 ± 0.35	2.25 ± 0.14	<0.50	12.22 ± 1.01	68.9	101.5	0.20 ± 0.01	7.4 ± 0.2
	20	0.98	4.53 ± 0.13	13.24 ± 1.14	2.34 ± 0.12	7.49 ± 0.22	1.14 ± 0.24	2.54 ± 1.10	<0.50	0.51 ± 0.22	97.5	106.2	0.19 ± 0.01	7.6 ± 0.1
	40	0.98	2.75 ± 0.16	6.55 ± 0.70	3.07 ± 0.23	9.10 ± 1.11	0.86 ± 0.24	1.36 ± 0.59	<0.50	2.14 ± 0.09	89.3	88.9	0.21 ± 0.04	7.4 ± 0.0
	0	5.4	4.34 ± 0.48	2.54 ± 0.46	1.87 ± 0.05	4.44 ± 1.51	2.27 ± 0.00	<0.50	<0.50	14.35 ± 0.47	28.3	106.7	0.16 ± 0.03	7.2 ± 0.2
	10	5.4	3.84 ± 0.54	9.35 ± 1.07	2.06 ± 0.01	4.74 ± 0.58	1.05 ± 0.20	3.06 ± 0.83	<0.50	4.87 ± 0.38	75.7	94.8	0.15 ± 0.02	7.7 ± 0.2
	20	5.4	2.89 ± 0.09	5.05 ± 1.33	2.79 ± 0.20	8.62 ± 0.28	0.34 ± 0.02	1.36 ± 0.04	<0.50	0.91 ± 0.23	95.5	72.9	0.17 ± 0.02	7.7 ± 0.1
	40	5.4	2.59 ± 0.04	5.31 ± 1.06	3.10 ± 0.28	9.91 ± 0.16	1.12 ± 0.20	2.98 ± 0.87	<0.50	3.27 ± 0.44	83.7	92.4	0.14 ± 0.0	7.8 ± 0.2

**Table 6:** Fermentation kinetics of valine (20 mM) in the presence of thiosulfate (20 mM) by *Thermoanaerobacter* strain AK85. Values represent the average of triplicate measures ± standard deviation.

Time (h)	Analyte (mmol/L)							% AA degraded	Carbon balance (%)	OD
	Ethanol	2-methyl-1-propanol	Acetate	2-methyl-1-propionate	H_2_	S_2_O_3_	Val			
4	3.37 ± 1.35	0.00 ± 0.00	2.36 ± 1.59	0.06 ± 0.00	0.32 ± 0.02	20.00 ± 0.00	20.00 ± 0.00	0.0	100.8	0.35 ± 0.09
8	3.54 ± 0.28	0.00 ± 0.00	1.10 ± 0.03	0.15 ± 0.19	0.45 ± 0.13	18.53 ± 1.01	20.00 ± 0.00	0.0	101.5	0.36 ± 0.04
12	5.87 ± 1.62	0.23 ± 0.01	1.15 ± 0.10	0.07 ± 0.01	1.07 ± 0.28	16.49 ± 0.77	20.00 ± 0.00	0.0	102.5	0.41 ± 0.02
18	6.76 ± 1.84	0.26 ± 0.01	1.69 ± 0.13	0.24 ± 0.04	1.14 ± 0.22	13.61 ± 0.75	20.00 ± 0.00	0.0	103.8	0.45 ± 0.04
24	7.43 ± 0.24	0.29 ± 0.03	1.97 ± 0.17	0.47 ± 0.06	1.59 ± 0.40	5.15 ± 0.78	20.00 ± 0.00	0.0	102.6	0.51 ± 0.12
30	7.19 ± 0.27	0.31 ± 0.02	1.91 ± 0.06	0.80 ± 0.16	1.44 ± 0.18	0.23 ± 0.04	19.41 ± 1.14	3.0	99.7	0.34 ± 0.06
36	6.90 ± 0.58	0.33 ± 0.03	1.97 ± 0.08	1.03 ± 0.17	2.48 ± 0.37	0.00 ± 0.00	18.58 ± 1.17	7.1	101.2	0.33 ± 0.07
48	6.58 ± 0.30	0.37 ± 0.07	2.04 ± 0.16	1.84 ± 0.40	2.23 ± 0.63	0.00 ± 0.00	18.03 ± 1.21	9.8	96.6	0.32 ± 0.08
60	7.39 ± 0.72	0.54 ± 0.09	2.05 ± 0.06	2.53 ± 0.32	1.94 ± 0.31	0.00 ± 0.00	16.25 ± 1.04	18.8	100.5	0.34 ± 0.05
72	7.42 ± 0.06	0.54 ± 0.11	2.08 ± 0.14	3.78 ± 0.68	2.31 ± 0.49	0.00 ± 0.00	15.78 ± 0.51	21.1	96.9	0.31 ± 0.10
120	3.00 ± 0.10	1.47 ± 0.11	2.37 ± 0.19	9.46 ± 1.03	1.83 ± 0.42	0.00 ± 0.00	8.44 ± 1.41	57.8	101.0	0.22 ± 0.03
168	1.54 ± 0.12	4.34 ± 0.24	3.78 ± 0.15	14.37 ± 0.82	1.07 ± 0.37	0.00 ± 0.00	1.49 ± 0.32	92.6	100.8	0.16 ± 0.04

**Table 7:** Fermentation kinetics of isoleucine (20 mM) in the presence of thiosulfate (20 mM) by *Thermoanaerobacter* strain AK85. Values represent the average of triplicate measures ± standard deviation.

Time (h)	Analyte (mmol/L)							% AA degraded	Carbon balance (%)	OD
	Ethanol	2-methyl-1-butanol	Acetate	2-methyl-1-butyrate	H_2_	S_2_O_3_	Ile			
4	6.96 ± 1.01	0.00 ± 0.00	0.69 ± 0.19	0.19 ± 0.04	0.17 ± 0.05	19.17 ± 0.57	20.00 ± 0.00	0.0	100.9	0.29 ± 0.04
8	3.27 ± 1.22	0.00 ± 0.00	0.84 ± 0.02	0.17 ± 0.02	0.63 ± 0.19	18.27 ± 1.31	20.00 ± 0.00	0.0	100.9	0.30 ± 0.08
12	4.92 ± 0.19	0.00 ± 0.00	1.40 ± 0.19	0.29 ± 0.15	1.43 ± 0.21	13.34 ± 1.13	20.00 ± 0.00	0.0	101.5	0.34 ± 0.06
18	4.22 ± 0.99	0.00 ± 0.00	1.79 ± 0.16	0.32 ± 0.02	1.72 ± 0.43	13.01 ± 1.36	20.00 ± 0.00	0.0	101.6	0.39 ± 0.05
24	5.16 ± 0.27	0.00 ± 0.00	1.94 ± 0.19	0.49 ± 0.10	1.63 ± 0.38	11.29 ± 0.41	20.00 ± 0.00	0.0	102.5	0.42 ± 0.02
30	5.11 ± 0.34	0.55 ± 0.09	1.98 ± 0.05	0.73 ± 0.24	1.84 ± 0.55	5.58 ± 1.77	20.00 ± 0.00	0.0	106.4	0.41 ± 0.08
36	5.51 ± 0.25	0.46 ± 0.01	2.02 ± 0.17	0.97 ± 0.32	2.07 ± 0.20	0.51 ± 0.19	20.00 ± 0.00	0.0	107.2	0.38 ± 0.07
48	5.22 ± 0.21	0.53 ± 0.02	2.15 ± 0.11	1.85 ± 0.64	2.43 ± 0.42	0.00 ± 0.00	19.37 ± 1.25	3.1	108.8	0.37 ± 0.05
60	5.44 ± 0.33	0.78 ± 0.01	2.03 ± 0.03	1.56 ± 0.12	1.20 ± 0.38	0.00 ± 0.00	18.60 ± 1.05	7.0	104.7	0.40 ± 0.09
72	5.09 ± 0.21	1.07 ± 0.03	2.28 ± 0.23	4.05 ± 1.01	1.40 ± 0.23	0.00 ± 0.00	16.82 ± 1.15	15.9	109.7	0.33 ± 0.00
120	4.84 ± 0.12	1.83 ± 0.02	2.41 ± 0.13	10.39 ± 0.51	1.03 ± 0.13	0.00 ± 0.00	7.42 ± 0.73	62.9	98.2	0.38 ± 0.05
168	2.21 ± 0.07	5.62 ± 0.18	4.05 ± 0.07	14.82 ± 0.37	0.79 ± 0.05	0.00 ± 0.00	1.07 ± 0.32	94.7	107.6	0.15 ± 0.03

**Table 8:** Fermentation kinetics of leucine (20 mM) in the presence of thiosulfate (20 mM) by *Thermoanaerobacter* strain AK85. Values represent the average of triplicate measures ± standard deviation.

Time (h)	Analyte (mmol/L)							% AA degraded	Carbon balance (%)	OD
	Ethanol	3-methyl-1-butanol	Acetate	3-methyl-1-butyrate	H_2_	S_2_O_3_	Leu			
4	6.96 ± 1.01	0.00 ± 0.00	0.69 ± 0.19	0.19 ± 0.04	0.34 ± 0.12	19.17 ± 0.57	20.00 ± 0.00	0.0	100.9	0.49 ± 0.09
8	3.27 ± 1.22	0.00 ± 0.00	0.84 ± 0.02	0.17 ± 0.02	0.78 ± 0.33	18.27 ± 1.31	20.00 ± 0.00	0.0	100.9	0.43 ± 0.07
12	4.92 ± 0.19	0.00 ± 0.00	1.40 ± 0.19	0.29 ± 0.15	1.07 ± 0.24	13.34 ± 1.13	20.00 ± 0.00	0.0	101.5	0.52 ± 0.11
18	4.22 ± 0.99	0.00 ± 0.00	1.79 ± 0.16	0.32 ± 0.02	1.40 ± 0.47	13.01 ± 1.36	20.00 ± 0.00	0.0	101.6	0.55 ± 0.12
24	5.16 ± 0.27	0.00 ± 0.00	1.94 ± 0.19	0.49 ± 0.10	1.66 ± 0.23	11.29 ± 0.41	20.00 ± 0.00	0.0	102.5	0.54 ± 0.13
30	5.11 ± 0.34	0.55 ± 0.09	1.98 ± 0.05	0.73 ± 0.24	1.83 ± 0.56	5.58 ± 1.77	20.00 ± 0.00	0.0	106.4	0.47 ± 0.11
36	5.51 ± 0.25	0.46 ± 0.01	2.02 ± 0.17	0.97 ± 0.32	2.25 ± 0.38	0.51 ± 0.19	20.00 ± 0.00	0.0	107.2	0.50 ± 0.13
48	5.22 ± 0.21	0.53 ± 0.02	2.15 ± 0.11	1.85 ± 0.64	2.41 ± 0.47	0.00 ± 0.00	19.37 ± 1.25	3.1	108.8	0.46 ± 0.02
60	5.44 ± 0.33	0.78 ± 0.01	2.03 ± 0.03	1.56 ± 0.12	2.87 ± 0.69	0.00 ± 0.00	18.60 ± 1.05	7.0	104.7	0.48 ± 0.06
72	5.09 ± 0.21	1.07 ± 0.03	2.28 ± 0.23	4.05 ± 1.01	1.63 ± 0.43	0.00 ± 0.00	16.82 ± 1.15	15.9	109.7	0.38 ± 0.02
120	4.84 ± 0.12	1.83 ± 0.02	2.41 ± 0.13	10.39 ± 0.51	1.28 ± 0.18	0.00 ± 0.00	7.42 ± 0.73	62.9	98.2	0.40 ± 0.08
168	2.21 ± 0.07	5.62 ± 0.18	4.05 ± 0.07	14.82 ± 0.37	1.32 ± 0.21	0.00 ± 0.00	1.07 ± 0.32	94.7	107.6	0.22 ± 0.08

## 2 Experimental design. materials and methods

### 2.1 General methods

Yeast extract was obtained from Difco while all other reagents were acquired from Sigma-Aldrich. Nitrogen gas was acquired from AGA and contained less than 5 ppm O_2_.

### 2.2 Microorganism and cultivation

*Thermoanaerobacter* strain AK85 (KR007650) (previously referred to as strain J1) was from our culture collection and 16S rRNA analysis of the strain was performed previously as described by [2] and references therein.

*Thermoanaerobacter* strain AK85 was cultivated in serum bottles or Hungate tubes using the Basal Mineral (BM) medium prepared as previously described [3] using the Hungate technique [4], [5]. BM consisted of (per liter): NaH_2_PO_4_·2H_2_O (3.04 g), Na_2_HPO_4_·2H_2_O (5.43 g), NH_4_Cl (0.3 g), NaCl (0.3 g), CaCl_2_·2H_2_O (0.11 g), MgCl_2_ x 6H_2_O (0.1 g), yeast extract (2.0 g), resazurin (1 mg), trace element solution (1 mL), vitamin solution 1 mL, Na_2_S x 9H_2_O (0.3 g), and NaHCO_3_ (0.8 g). The trace element solution was prepared as follows on a per liter basis: FeCl_2_·4H_2_O (2.0 g) was dissolved in 10 mL of 25% v/v HCl to which 400 mL of dH_2_O and Na_2_EDTA·2H_2_O (0.5 g) was added; the following reagents were then added sequentially: CuCl_2_ (0.03 g), H_3_BO_3_ (0.05 g), ZnCl_2_ (0.05 g), MnCl_2_·4H_2_O (0.05 g), (NH_4_)6Mo_7_O_24_·4H_2_O (0.05 g), AlCl_3_ (0.05 g), CoCl_2_·6H_2_O (0.05 g), Na_2_SeO_3_ (0.03 g), and Na_2_WO_4_·2H_2_O (0.03 g). Filled to a V_f_ of 1 L; 1 mL aliquots were frozen at −40 °C prior to use. The vitamin solution was prepared according to DSM 141 and consisted of (on a per L basis): biotin (2.0 mg), folic acid (2.0 mg), pyridoxine-HCl (10.0 mg), thiamine-HCl·2H_2_O (5.0 mg), nicotinic acid (5.0 mg), D-Ca-pantothenate (5.0 mg), cobalamin (0.1 mg), *p*-aminobenzoic acid (5.0 mg), and lipoic acid (5.0 mg); the vitamin solution was stored at −20 °C prior to use. The medium was assembled by adding the 1 M phosphate buffer (pH 7.0) and yeast extract to distilled water containing resarzurin and boiled for 10–15 min until pink; the solution was cooled to ambient temperature under a stream of nitrogen (<5 ppm O_2_). The mixture was then transferred to serum bottles and autoclaved (121 °C) for 60 min. All other components of the medium were added separately through filter (0.45 μm) sterilized solutions. Substrates were provided at a concentration 20 mM unless specifically stated otherwise stated. All fermentations were done at 65 °C and at pH of 7.0 with a liquid-gas (L-G) ratio of 1:1 without agitation except when stated otherwise. All inoculations were performed using cultures taken from the exponential growth phase using an inoculation volume of 2% (v/v). Overnight stocks cultures of strain AK85 were cultivated on glucose (20 mM). All cultivations were performed as triplicates.

### 2.3 Amino acid substrate utilization with and without electron scavenging systems

The 20 proteogenic amino acids were cultivated as single substrates (20 mM) with or without the addition of thiosulfate (40 mM).

### 2.4 Effect of initial pH on isoleucine fermentation

To investigate the effect of pH on the end product profile from isoleucine, strain AK85 was cultivated in Hungate tubes (15 × 150 mm) in BM containing 20 mM of isoleucine and 20 mM of thiosulfate at pH ranging from pH 4.0 to 9.0 with 0.5 pH unit intervals. End products were determined after 14 days of incubation.

### 2.5 Effect of temperature on isoleucine fermentation

To investigate the effect of temperature on growth, strain AK85 was cultivated at 50 °C–85 °C in 5 °C intervals in Hungate tubes (15 × 150 mm) as otherwise described in section 2.4. End products were determined after 14 days of incubation.

### 2.6 Effect of liquid-gas phase ratio

Strain AK85 was cultivated in serum bottles (57 mL nominal volume) which were filled with either 4.5, 13.4, 26.5, 36.0, or 45.0 mL of BM medium containing isoleucine (20 mM) and thiosulfate (20 mM) to give L-G values of 0.09, 0.34, 0.98, 2.12, and 5.62, respectively. End products were quantified after 14 days of cultivation.

### 2.7 Effect of initial thiosulfate concentration

The effect of initial thiosulfate concentration on isoleucine (20 mM) degradation pattern was investigated with initial thiosulfate concentrations between 0 and 60 mM. The experiments were performed Hungate tubes (15 × 150 mm) as otherwise described in section 2.4. End products were determined after 14 days of incubation.

### 2.8 Effect of initial thiosulfate concentration and liquid-gas phase ratio

In one experiment both the effects of different L-G phase ratios as well as different initial thiosulfate concentrations were tested using isoleucine (20 mM) as the substrate. Three different L-G phase ratios (0.05, 0.98, 5.40) were used and thiosulfate concentrations ranged from 0 to 40 mM.

### 2.9 Kinetic experiments

Fermentation kinetics of leucine, isoleucine, and valine (20 mM) in the presence of thiosulfate (20 mM) were conducted in 125 mL serum bottles at a L-G ratio of 0.98 over a period of 7 days.

### 2.10 Analytical methods

Hydrogen, volatile fatty acids and alcohols were quantified by gas chromatography as described earlier [6]. Thiosulfate, hydrogen sulphide, and amino acids were analysed as previously described [7]. Optical density (OD) was measured at 600 nm with a Shimadzu UV-1800 UV–Visible spectrophotometer with cuvetted (*l* = 1 cm) against a water blank.
