# Supplementary material for: Dataset describing the amino acid catabolism of Thermoanaerobacter strain AK85: The influence of culture conditions on end product formation
Source: Data Brief. 2019 Apr 23;24:103938. doi: 10.1016/j.dib.2019.103938 (PMC6503125; doi:10.1016/j.dib.2019.103938)
Supplement: Multimedia component 1 [file mmc1.docx]

Conflict of interest.

The authors claim there are no conflict of interest concerning the material presented in the submission of the manuscript „Dataset Describing the Amino Acid Catabolism of *Thermoanaerobacter* strain AK85: the Influence of Culture Conditions on End Product Formation“.
